# Protein Glutathionylation in the Pathogenesis of Neurodegenerative Diseases

**DOI:** 10.1155/2017/2818565

**Published:** 2017-12-31

**Authors:** Sun Joo Cha, Hayoung Kim, Hyun-Jun Choi, Sanghyun Lee, Kiyoung Kim

**Affiliations:** ^1^Soonchunhyang Institute of Medi-Bio Science, Soonchunhyang University, Cheonan 31151, Republic of Korea; ^2^Department of Medical Biotechnology, Soonchunhyang University, Asan 31538, Republic of Korea

## Abstract

Protein glutathionylation is a redox-mediated posttranslational modification that regulates the function of target proteins by conjugating glutathione with a cysteine thiol group on the target proteins. Protein glutathionylation has several biological functions such as regulation of metabolic pathways, calcium homeostasis, signal transduction, remodeling of cytoskeleton, inflammation, and protein folding. However, the exact role and mechanism of glutathionylation during irreversible oxidative stress has not been completely defined. Irreversible oxidative damage is implicated in a number of neurological disorders. Here, we discuss and highlight the most recent findings and several evidences for the association of glutathionylation with neurodegenerative diseases and the role of glutathionylation of specific proteins in the pathogenesis of neurodegenerative diseases. Understanding the important role of glutathionylation in the pathogenesis of neurodegenerative diseases may provide insights into novel therapeutic interventions.

## 1. Glutathione

Glutathione (GSH) is abundant in all cells as a low molecular weight thiol with intracellular concentrations usually ranging from approximately 1 to 10 mM *in vivo* [1, 2]. Most of the cellular GSH exists in the cytosol, and the remaining in organelles including mitochondria, nuclear matrix, and peroxisomes [3]. GSH can be converted to the oxidized form, glutathione disulfide (GSSG), which is then converted back into GSH by the nicotinamide adenine dinucleotide phosphate- (NADPH-) dependent glutathione disulfide reductase [4]. GSH is oxidized nonenzymatically to GSSG by electrophilic substrates through the cysteine residue. Various enzymes, glutathione peroxidase (Prx), glutaredoxin (Grx), and thioredoxin (Trx), participate in GSH redox homeostasis, which is defined by the GSH concentration divided by the GSSG concentration, while glutathione reductase ensures that GSH remains in a reduced form and maintains a high GSH/GSSG ratio in the cytosol. The reduced form of GSH is the predominant form of the total GSH pool [5, 6]. The intracellular redox state, including the level of GSH and GSSG, and the GSH/GSSG ratio are an important marker of oxidative stress and cellular health [7, 8].

GSH is synthesized in the cytoplasm; it is translocated to different organelle-specific functions. It may be implicated in organelle-specific functions related to the regulation of redox homeostasis. The redox state of GSH in the endoplasmic reticulum (ER) possesses more oxidative than that in cytosol for maintaining and promoting the folding and posttranslational modifications of the proteins. GSSG is the source of oxidizing power that ensures the functional conformation of native polypeptides by the formation of required intramolecular disulfide linkages in the ER [9, 10]. Mitochondrial GSH is transported from the cytoplasm by the activity of a specific carrier protein [11, 12]. Mitochondrial GSH is the primary defense against oxidative stress in mitochondrial membrane to ensure the reduction of hydroperoxides on phospholipids. Furthermore, nuclear GSH has been postulated to play a role in the control of cell cycling, DNA replication, chromatin compaction, epigenetics, and activity of transcription factors [13–15].

The major functions of GSH are acting as the indicator of the cellular redox state, antioxidant defense, and the storage and transport of cysteine [16, 17]. The antioxidant reactions of GSH are carried out by glutathione peroxidases, which reduce hydrogen peroxide and lipid hydroperoxides by oxidizing GSH to GSSG [18]. GSH can also form conjugates with a great variety of electrophilic compounds, by the action of glutathione transferases (GSTs) [19, 20]. Furthermore, lymphocytes and intestinal epithelial cells require a sufficient intracellular GSH concentration to maintain a reduced redox potential for proliferation [21]. Moreover, GSH is crucial for the activation of lymphocytes [6]. Although GSH exerts antioxidant activities through antioxidant enzymes, it also possesses protective functions against oxidative damage by nonenzymatic free radical scavenging. Since oxidative stress is involved in the pathogenesis of many human diseases including neurodegenerative diseases, GSH has been shown to play a defensive role under the oxidative stress conditions in these diseases [6, 22]. Especially, dysfunction of GSH metabolism is common in several neurodegenerative diseases showing GSH depletion [23, 24]. GSH depletion would induce oxidative stress in the brain, leading to neurodegeneration [23]. Oxidative stress is involved in age-related neurodegenerative diseases. However, the precise molecular mechanism of GSH depletion is still unclear and thus considered to be worthwhile for further study. Furthermore, a therapeutic strategy to recover neuronal GSH contents in the brain is a critical treatment for GSH depletion-related neurodegenerative diseases. However, no therapeutic drugs are available for increasing GSH contents in the brain at present.

## 2. Protein Modification: Glutathionylation

Under normal physiological conditions and oxidative stress, many redox modifications can occur. Especially, cysteine thiols (-SH) of proteins can become targets of reactive oxygen species (ROS) and modulators including glutaredoxins and glutathione transferases. It can cause a chain of modifications in their oxidation state, which can conclude the fate of particular proteins. Redox modifications can enable the cysteine thiols of protein to maintain and tolerate the irreversible and reversible posttranslational modifications during oxidative stress.

Protein palmitoylation is an important reversible lipid modification in which one or more cysteine thiols on a substrate are modified to form a thioester with a palmitoyl group [25]. The reversible cycles of palmitoylation and depalmitoylation play a critical role in intercompartment shuttling of modified substrate proteins under conditions that alter the cellular redox state [26]. Protein nitrosylation is also a redox-mediated modification that regulates target protein functions by covalent binding of nitric oxide with a cysteine thiol on the target proteins. Nitrosylation can regulate ion channels and protein activity of metabolic enzymes, protein kinases, phosphatases, oxidoreductases, and transcription factors [27].

Protein glutathionylation is considered as a defense mechanism to protect proteins from oxidative states that leads to irreversible damage and is the reversible posttranslational modification on cysteine thiol groups of the protein through the disulfide bond with GSH [16]. Glutathionylation can occur through either nonenzymatic or enzymatic reactions. Nonenzymatic formation of glutathionylation depends on the availability of GSH/GSSG. Glutathionylation is readily reversible via the release of GSH from the cysteine residues in the target proteins by Grx and Trx [28, 29]. Several target proteins with potential glutathionylation have been identified [30]. For instance, glyceraldehyde-3-phosphate dehydrogenase (GAPDH) and *α*-ketoglutarate dehydrogenase complex (KGDHC), which function in the glycolytic pathway and Krebs cycle, respectively, are regulated by glutathionylation. GAPDH is inactivated via glutathionylation on Cys149 in endothelial cells following exposure to oxidative stress [31, 32]. Mitochondrial KGDH activity is also inactivated by glutathionylation in rat liver following exposure to hydrogen peroxide. Furthermore, the E2 subunit of KGDHC is reversibly glutathionylated on the covalently attached cofactor lipoic acid [33]. In addition, glutathionylation regulates cell proliferation and survival through activation of the apoptotic pathway. Caspase-3 is modified by glutathionylation, and Grx regulates tumor necrosis factor (TNF)-*α*-induced apoptosis through glutathionylation of caspase-3 [34]. For example, protein tyrosine phosphatase 1 B (PTP1B) can easily undergo oxidation during oxidative conditions. Glutathionylation of the active site Cys215 modulates the PTP1B activity [35–38]. Furthermore, nuclear factor kappa B (NF-*κ*B) is a transcription factor that upregulates the expression of many genes involved in inflammation, cell proliferation, and defense against apoptosis [39]. NF-*κ*B can itself be directly glutathionylated. The glutathionylation of NF-*κ*B subunit p50 at Cys62, which lies in the DNA-binding domain, has been shown to induce the reversible blocking its DNA-binding activity [40]. Another group reported that Cys179 of the inhibitory *κ*B kinase (IKK) *β* subunit is a target of glutathionylation during oxidative stress. Glutathionylation of IKK*β* is reversed by Grx, which rescues the activity of kinase [41]. Moreover, p53 is a tumor suppressor protein and acts as a transcription factor that regulates cell cycle and apoptosis [42]. It is also glutathionylated on Cys124, Cys141, and Cys182 in the DNA-binding region in human malignant glioblastoma and colon carcinoma cells. Glutathionylated p53 at Cys141 loses the ability to recognize the consensus DNA sequence [43, 44]. Cellular calcium levels are regulated by the transport systems of calcium, the sarcoplasmic endoplasmic reticulum calcium ATPase (SERCA), and the ryanodine receptor (RyR) in endoplasmic reticulum (ER). SERCA, the system of calcium uptake in ER, is activated by glutathionylation on Cys674 during nitrosative stress [45]. In contrast to the function of SERCA, RyR is an ER calcium release channel to the cytosol. NADPH oxidase-dependent RyR1 glutathionylation stimulates to faster calcium release in muscles [46]. In addition, RyR2 was shown to be glutathionylated in rat model of cerebral ischemia [47]. Cytoskeletal structure in cells is also regulated by glutathionylation. Actin is found in almost all cell types and acts in cytoskeletal organization. Glutathionylation inhibits actin polymerization for regulation of the cytoskeleton structure and functions in cell spreading and disassembly of actin-myosin complexes during cell adhesion [48].

Thus, as discussed above, glutathionylation possesses several biological functions such as regulation of metabolism pathways, calcium homeostasis, modulation of cell signaling, apoptotic pathways, remodeling of cytoskeletal organization, inflammation, and protein folding. However, the exact role and mechanism of glutathionylation during oxidative stress have not been completely defined. Although significant progress has been made in deciphering the biological roles of protein glutathionylation in cells, several critical questions regarding the selection of target and the tight regulation of glutathionylation remain.

## 3. Protein Glutathionylation in Neurodegenerative Diseases

Neurodegenerative diseases involve some common pathological and pathogenesis characteristics such as progression of neuron loss, aggregation of misfolded proteins, mitochondrial dysfunction, increased removal of iron, and neuronal cell death due to the overexposure to reactive nitrogen species (RNS) and reactive oxygen species (ROS) [49–51]. The most typical neurodegenerative diseases include Alzheimer's disease (AD), Parkinson's disease (PD), amyotrophic lateral sclerosis (ALS), and Huntington's disease (HD). Each disease involves specific proteins that contribute to the onset or progression of the disease. However, the critical factors underlying the pathogenesis of these neurodegenerative diseases still remain poorly understood. Recent various studies have suggested that the glutathionylation of specific proteins could contribute to the onset or progression of these neurodegenerative diseases [52–54]. The relationships between glutathionylation and each of these neurodegenerative diseases are described below, and the glutathionylation of specific proteins is also described (Table 1). The target proteins, molecular mechanisms, and functional importance of glutathionylation have been described in several reviews [52, 54]. Nevertheless, there is a growing interest for glutathionylation and the number of glutathionylated proteins started to increase recently in pathogenesis of neurodegenerative diseases. This review describes the most recent findings on the novel glutathionylated proteins and their functions in neurodegenerative diseases.

### 3.1. Alzheimer's Disease

Neurodegenerative diseases exhibit common pathological and pathogenesis characteristics such as the progression of neuron loss. AD is a progressive neurodegenerative disease in which there is a decrease in memory and recognition ability. There are some specific characteristics in AD pathology, including the formation of intracellular neurofibrillary tangles that result in accumulation of hyperphosphorylated tau proteins, disruption of axonal transport, and amyloid-*β* (A*β*) peptide aggregation surrounded by dying neurites called senile plaques [55, 56]. A*β* is associated with mitochondrial dysfunction, increased calcium levels, and breakdown of the membranes. AD follows after the accumulation of A*β* induced by oxidative stress [57]. Specific proteins such as GAPDH, which participate in the glycolytic pathway, exhibit increased glutathionylation in AD inferior parietal lobule compared with age-matched controls. Glutathionylation inhibits GAPDH activity in AD patients. *α*-Enolase is also glutathionylated in AD brain and that this is associated with a decreased activity [58]. Furthermore, it has been shown that GSH/GSSG ratio decreases in the brain of aged rats [59], and there is oligomerization and aggregation of glutathionylated p53 in the inferior parietal lobule of AD patients. In addition, selective glutathionylation of p53 monomers and dimers has been found in AD brain [60]. Tau protein has been shown to be involved in cytoskeletal protein and microtubule-associated protein [61, 62]. Glutathionylated tau protein can be detected through mass spectrometry, and its function can be altered by polymerization of 3-repeat tau [63, 64]. Moreover, actin functions in the maintenance of cytoskeletal structure. Actin glutathionylation has been implicated in neurological disease. For example, fibroblasts from Freidreich's ataxia patients showed increased actin glutathionylation [65]. AD brain samples also show increased actin oxidation [66, 67]. As already mentioned above, there are increasing evidence from numerous studies that correlate the glutathionylation of specific proteins and the pathogenic mechanism of AD. These results further emphasize the involvement of glutathionylation in the pathogenesis of AD. However, further studies needed to determine whether regulation of specific proteins by glutathionylation directly contributes to AD pathology.

### 3.2. Parkinson's Disease

PD is a common neurodegenerative disorder, which occurs due to the loss of dopaminergic neurons in the *substantia nigra* of the midbrain [68]. PD is classified into two kinds: sporadic, for which the specific cause is unknown, and familial, which is a heritable disease. Oxidative stress mediates the pathogenesis of sporadic PD linked with aging [69]. The familial PD is caused by genetic mutations in specific proteins such as *PARK2* (Parkin), *SNCA* (*α*-synuclein), *PINK1* (Pink1), and *PARK7* (DJ-1) [70–72]. These proteins regulate the cellular signaling pathways involved in respiration and transport system, mitochondrial dynamics, calcium homeostasis, ROS production, autophagy, and apoptosis [73–77]. Although mutations in these proteins are known to cause PD, oxidative modifications of these proteins may also contribute to the pathogenesis of PD [78]. The protein parkin harbors cysteine residues, which are susceptible to oxidative modification, and treatment with hydrogen peroxide has been shown to cause diminished Parkin activity. The deposits of misfolded *α*-synuclein act as a core for the association with other proteins. These deposits, which consist of an ubiquitin-proteasome system and intraneuronal inclusions, are called Lewy plaques. *α*-Synuclein is localized in the synaptic vesicles and on the cell membranes of nervous tissue [79]. Posttranslational modification of *α*-synuclein including phosphorylation and nitrosylation can cause misfolding and deposition of the protein [80]. DJ-1 functions in neuroprotection against oxidative stress by serving as a redox sensor under reducing condition [81]. Recently, Mieyal's group reported evidence that a posttranslational mechanism for regulation of DJ-1 content involving reversible glutathionylation. They showed that Grx regulates DJ-1 protein content *in vivo*. Furthermore, they found that DJ-1 is susceptible to glutathionylation *in vitro* and *in vivo* [82]. These results suggest that glutathionylation of DJ-1 contributes the regulation of its protein degradation mechanism. In a mouse model of PD, treatment of the neurotoxin 1-methyl-4-phenyl-1,2,3,6-tetrahydropyridine (MPTP), which inhibits the mitochondrial complex I and induces the Grx activity increase in the brain, provoked selective damage to dopaminergic neurons. Furthermore, when Grx was downregulated, MPTP-induced complex I inhibition was not reserved [83]. In addition, glutathionylation of Ndusf1 and Ndufv1 subunits in mitochondrial complex I leads to a decrease in the activity of complex I [84]. Despite the limitations of *in vitro* studies, one can suggest from these observations that Grx may serve to deglutathionylate the subunit of mitochondrial complex I and play an important role in maintaining mitochondrial complex I activity in sporadic PD. In mice treated with MPTP to simulate PD, glutathionylation of mitochondrial NADP^+^-dependent isocitrate dehydrogenase has been reported [85]. In *Drosophila* PD model induced by loss of *parkin* gene, glutathionylation levels of ATP synthase *β* subunit are decreased and restored by expressing GST omega (GstO) [86]. Glutathionylation of ATP synthase *β* subunit by GstO regulates mitochondria F1F0-ATP synthase activity and is important for restoration of mitochondrial function in *Drosophila* PD model.

### 3.3. Amyotrophic Lateral Sclerosis

ALS, also known as the Lou Gehrig's disease, is a devastating adult-onset neurodegenerative disease characterized by progressive degeneration of the motor neurons. ALS leads to gradual muscle weakness, eventually leading to fatal muscle atrophy and paralysis, and death within 3~5 years of disease onset [87]. ALS can be classified into sporadic ALS, caused by unknown pathogenesis, and familial ALS, caused by genetic defects directly linked to pathogenesis, including superoxide dismutase 1 (SOD1), transactive response DNA-binding protein (TDP-43), fused in sarcoma/translocated in liposarcoma protein (FUS/TLS), and TATA-box binding protein-associated factor 15 (TAF15). Mutant forms of TDP-43, FUS, are toxic to neurons and that mislocalization of these proteins to the cytoplasm is critical for disease pathogenesis [88, 89]. SOD1-positive inclusions have been detected in motor neurons in ALS patients [90]. Protein aggregations are a hallmark of all types of ALS and are found in motor neurons [91–93]. Although potential regulators of protein aggregation and mislocalization have been identified in ALS, the exact mechanisms of pathogenesis have not been fully investigated. It has been reported that oxidative modification of Cys111 in SOD1 such as glutathionylation is liable to decrease its enzymatic activity [94, 95]. Peroxidized SOD1 at Cys111 is detected in the spinal motor neurons of G93A mutant SOD1 transgenic mice [96]. In addition, human SOD1 harbors four cysteine residues. Among these cysteine residues, Cys57 and Cys146 create the disulfide bridge [97, 98]. Reduction of disulfide bond between Cys57 and Cys146 makes human SOD1 liable to misfold, resulting in monomerization [99]. These results show the pathogenic significance of cysteine residues for aggregate formation to acquire neuronal toxicity. Although the posttranslational modification of SOD1 at cysteine residues may relate to inactivation and monomerization, further investigation will be needed.

### 3.4. Huntington's Disease

HD is a neurodegenerative disorder characterized by loss of motor control and recognition ability. HD is caused by abnormal CAG triplet expansions, which encodes a poly-glutamine repeat at the N-terminus of the huntingtin (HTT) protein [100]. An abnormal expansion of glutamine leads to formation of toxic oligomerization and aggregation of HTT [101]. HD results due to many factors including changes in calcium signaling pathway, IGF signaling, vesicle transport, and ER maintenance [102]. Mutants in HTT cannot modulate the calcium signaling in the mitochondria, thus decreasing the calcium concentration [103]. Moreover, toxicity by glutamate contributes to the death of neuron cells and is mediated by ROS and GSH loss [104]. In 2015, Professor So's group revealed the increased Ca^2+^-permeable transient receptor potential cation (TRPC) channel member 5 (TRPC5) in the striatum of transgenic mice and patient with HD. *In vitro* studies further demonstrated that TRPC5 is glutathionylated at Cys176 and Cys178 by oxidative stress such as GSH loss. This results inactivation of the calcium channel, leading to an influx of calcium into the cells and eventually death of neurons [105]. TRPC5 glutathionylation occurs at higher levels in HD models and leads to neurodegeneration. These results strongly suggest that the activation of TRPC5 through glutathionylation is novel pathological mechanism that regulates neuronal damage in HD.

## 4. Therapeutic Implications and Conclusion

**Figure 1:**
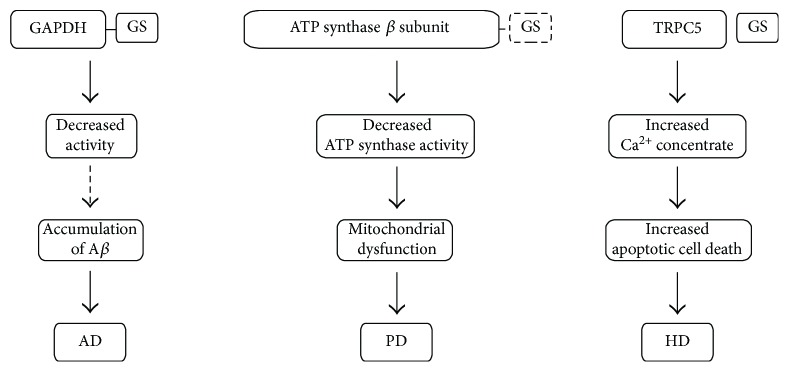
Proposed mechanisms for specific-protein glutathionylation/deglutathionylation in the pathogenesis of several neurodegenerative diseases. Glutathionylation of GAPDH can lead to a decrease in the enzymatic activity. This process can contribute to accumulation of A*β* and induce AD. Additionally, glutathionylation of TRPC5 triggers increased calcium uptake leading to apoptotic cell death and contributes to the pathogenesis of HD. Decreased glutathionylation of ATP synthase *β* subunit can inhibit enzymatic activity of mitochondrial ATP synthase. This alteration affects mitochondrial function and can contribute to the development of PD.

Protein glutathionylation is one of the important posttranslational modifications that regulate various cellular processes by regulating protein functions and prevents irreversible oxidation of cysteine thiol in the target proteins. Numerous pieces of evidence suggest that glutathionylation of specific proteins is associated with a number of neurodegenerative diseases, including AD, PD, ALS, and HD. Protein glutathionylation can occur in response to oxidative damage and other stress and may contribute to neurodegeneration by dysregulating several cellular processes (Figure 1). Recently, several glutathionylated proteins have been identified and they can be changed in disease conditions [106]. However, further studies are required to determine whether the regulation of several other disease-related proteins by glutathionylation contributes to neuropathological conditions.

Since protein glutathionylation is likely to regulate protein functions and cellular signaling pathways that play a critical role in pathogenesis of neurodegenerative diseases, it is an important therapeutic target for drug development. However, no therapeutic drugs are available for modulating protein glutathionylation in the progression of neurodegenerative diseases. The potential involvement of glutathionylation in human neurodegenerative diseases highlights the need of a better understanding of how diseases are caused by glutathionylation of specific proteins and if they are targets for prevention of neurodegenerative disease.

## Figures and Tables

**Table 1:** Summary of the glutathionylated proteins involved in neurodegenerative diseases.

Disease	Target protein of glutathionylation	Effect of glutathionylation	Reference
AD	Glyceraldehyde-3-phosphate dehydrogenase	Inhibition of *α*-enolase activity	[58]
	p53	Possibly prevention of its tetramer formation	[60]
	*α*-Enolase	Inactivation	[58]

PD	Mitochondrial NADP^+^-dependent isocitrate dehydrogenase	Inhibition of its activity	[85]
	ATP synthase *β* subunit	Possible regulation of complex V assembly	[86]

HD	TRPC5	Activation of Ca^2+^ channel	[105]
